# Comparative Analysis of META and SALT Disaster Triage in an Adult Trauma Population: A Retrospective Observational Study

**DOI:** 10.1017/S1049023X24000098

**Published:** 2024-04

**Authors:** Gawin Tiyawat, J. Marc Liu, Thongpitak Huabbangyang, Cesar Luis Roza-Alonso, Rafael Castro-Delgado

**Affiliations:** 1. Department of Disaster and Emergency Medical Operation, Faculty of Science and Health Technology, Navamindradhiraj University, Bangkok, Thailand; 2. Department of Emergency Medicine, Medical College of Wisconsin, Milwaukee, Wisconsin USA; 3. Health Service of the Principality of Asturias (SAMU-Asturias), Health Research Institute of the Principality of Asturias (Research Group on Prehospital Care and Disasters, GIAPREDE), Oviedo, Spain; 4. Department of Medicine, Oviedo University, Oviedo, Spain; 5. RINVEMER-SEMES (Research Network on Prehospital Care-Spanish Society of Emergency Medicine), Madrid, Spain

**Keywords:** data accuracy, triage, disasters, mass-casualty incidents, mass-casualty triage, prehospital care

## Abstract

**Background:**

Medical professionals can use mass-casualty triage systems to assist them in prioritizing patients from mass-casualty incidents (MCIs). Correct triaging of victims will increase their chances of survival. Determining the triage system that has the best performance has proven to be a difficult question to answer. The Advanced Prehospital Triage Model (Modelo Extrahospitalario de Triaje Avanzado; META) and Sort, Assess, Lifesaving Interventions, Treatment/Transport (SALT) algorithms are the most recent triage techniques to be published. The present study aimed to evaluate the META and SALT algorithms’ performance and statistical agreement with various standards. The secondary objective was to determine whether these two MCI triage systems predicted patient outcomes, such as mortality, length-of-stay, and intensive care unit (ICU) admission.

**Methods:**

This retrospective study used patient data from the trauma registry of an American College of Surgeons Level 1 trauma center, from January 1, 2018 through December 31, 2020. The sensitivity, specificity, and statistical agreement of the META and SALT triage systems to various standards (Revised Trauma Score [RTS]/Sort Triage, Injury Severity Score [ISS], and Lerner criteria) when applied using trauma patients. Statistical analysis was used to assess the relationship between each triage category and the secondary outcomes.

**Results:**

A total of 3,097 cases were included in the study. Using Sort triage as the standard, SALT and META showed much higher sensitivity and specificity in the Immediate category than for Delayed (Immediate sensitivity META 91.5%, SALT 94.9%; specificity 60.8%, 72.7% versus Delayed sensitivity 28.9%, 1.3%; specificity 42.4%, 28.9%). With the Lerner criteria, in the Immediate category, META had higher sensitivity (77.1%, SALT 68.6%) but lower specificity (61.1%) than SALT (71.8%). For the Delayed category, SALT showed higher sensitivity (META 61.4%, SALT 72.2%), but lower specificity (META 75.1%, SALT 67.2%). Both systems showed a positive, though modest, correlation with ISS. For SALT and META, triaged Immediate patients tended to have higher mortality and longer ICU and hospital lengths-of-stay.

**Conclusion:**

Both META and SALT triage appear to be more accurate with Immediate category patients, as opposed to Delayed category patients. With both systems, patients triaged as Immediate have higher mortality and longer lengths-of-stay when compared to Delayed patients. Further research can help refine MCI triage systems and improve accuracy.

## Introduction

A disaster or mass-casualty incident (MCI) occurs when the medical needs of many patients exceed the available medical resources.^1^ Cases of MCIs are more frequent than people realize, according to a recent population-based epidemiological study, and should therefore be taken into account when formulating response plans.^2,3^ In order to improve outcomes when an MCI event occurs, the focus changes from providing the best care possible for each patient to offering the best possible treatment for the greatest number of people.^1,4^ Thus, a medical disaster event necessitates quick and effective triage. This triage is defined as a classification of patients according to severity to determine the priority of treatment and evacuation.^5^

In order to properly allocate limited resources, medical professionals can prioritize MCI patients with the guidance of mass-casualty triage systems. Victims of MCIs may have a better chance of surviving and achieving a better outcome if they are properly triaged.^6^ Currently, various countries and regions have developed and implemented mass-casualty triage systems. It has proven challenging to determine which triage system is better in terms of accuracy and outcomes. The most recent triage methods to be published are the Advanced Prehospital Triage Model (META; Modelo Extrahospitalario de Triaje Avanzado) and Sort, Assess, Lifesaving Interventions, Treatment/Transport (SALT) algorithms. In this study, these two triage systems were chosen to be compared and analyzed.

In 2008, Lerner, et al introduced the SALT triage algorithm, a proposed national triage guideline based on the evidence and consensus opinion.^7^ This triage method aims to standardize the mass-casualty triage method used throughout the United States. The SALT triage method is intended to be applicable to both adults and children, and to be utilized in all-hazard situations. The SALT triage main steps include global sorting, individual assessment, basic lifesaving interventions, and assigning triage categories to prioritize treatment and/or transport.^7^ This triage is now recommended in the United States as being compliant with the Model Uniform Core Criteria, which define the characteristics/goals of a mass-casualty triage system.^8^ The SALT system has received the support of the American College of Emergency Physicians (ACEP; Irving, Texas USA), the American Trauma Society (ATS; Falls Church, Virginia USA), and the American College of Surgeons Committee on Trauma (ACS COT; Chicago, Illinois USA).^8^

In 2010, and updated in 2021, a Spanish triage method called META was designed to be implemented as prehospital triage in MCIs and disasters. Treatment triage (primary triage) and evacuation triage are the two key components of the META triage system. The treatment triage (using the Airway, Breathing, Circulation, Disability, Exposure [ABCDE] technique of Advanced Trauma Life Support) prioritizes on-scene care by assessing whether an urgent surgical examination is required: the so called “surgical patients.”^9,10^ Presently, it is widely used by Spanish Emergency Medical Services (EMS) systems, which use a physician-staffed EMS model.

Despite the fact that several triage methods have been developed and utilized, the lack of a definite, accepted gold standard for the “correct” triage category is a constraint to the advancement of the science of mass-casualty triage.^1^ The detailed metrics used to measure resource utilization and/or outcome vary from study to study. There is often no agreement between studies on how to characterize triage categories by outcome measures. In some studies, rather than evaluating the patient’s actual use of medical resources, the expected triage result as determined by an experienced expert is used to assess triage accuracy.^11–13^ It is therefore challenging for investigators to compare or analyze mass-casualty triage methods in an efficient manner. Thus, a uniform set of criteria defining each triage category based on the actual patient resource utilization and outcome was proposed by Lerner and colleagues in 2015. The intent was to standardize the “correct” triage category in order to conduct research on the accuracy of mass-casualty triage systems.^1^

Various studies have been conducted to estimate the accuracy of different types of triage systems, but, to the best of the authors’ knowledge, there is no comprehensive study on the comparison of different types of triage methods. The primary objective of this research is to evaluate the performance and statistical agreement of the META and SALT mass-casualty triage systems with a number of standards. The ability of the two MCI triage systems to predict patient outcomes (such as emergency department [ED] disposition, intensive care unit [ICU] admission and length-of-stay, hospital length-of-stay, morbidity, and blood product usage within 24 hours) is being examined as a secondary objective.

## Methods

### Study Design and Setting

This study was a retrospective data review of patients from the trauma database of Froedtert Hospital (Milwaukee, Wisconsin USA), which is an American College of Surgeons Level 1 trauma center. Froedtert Hospital serves as the primary academic tertiary-care medical center for an area with a population of over two million people with urban, suburban, and rural settings.^14^ The data in the trauma registry are validated in accordance with American College of Surgeons data standards. Automated checks are conducted using commercially available data software that is regularly updated to College standards. Errors detected are manually reconciled. In addition, 10% of cases are validated manually using the inter-rater reliability method. Both are standard techniques used for trauma registries across the industry. This study was conducted according to the Standards for the Reporting of Observation Studies in Epidemiology (STROBE) statement.^15^

### Selection Criteria

#### Eligibility Criteria

Patients aged 18 years and above and managed by Froedtert Hospital under trauma activation criteria from January 1, 2018 through December 31, 2020 were included.

#### Exclusion Criteria

Exclusion criteria were patients younger than 18 years, those presenting as interfacility transfers, and those with missing/incomplete data in the database.

### Data Gathering

The analyzed data fields were abstracted from the trauma registry by database managers who were not study investigators, and then collated into a separate and secured study database (Microsoft Excel; Redmond, Washington USA). The abstraction process removed all identifiers except for age. The principal investigator used the study database to verify all cases to ensure they met inclusion criteria. Baseline characteristics of studied cases included gender, age, injury type (blunt, penetrating, or burn), mechanism of injury, comorbidities, current smoker, alcohol use disorder, substance use disorder, anticoagulant therapy, and steroid use (Table 1). Hospital disposition characteristics included post-ED disposition, discharge status, ICU length of stay (days), use of mechanical ventilation (days), and total hospital length of stay (days); Table 2.

**Table 1.** Patient Characteristics

Characteristics	Total	Male	Female	P Value
All Patients, N (%)	3,097 (100.0)	1,822 (58.8)	1,275 (41.2)	
Age, Years (SD)	55.5 (SD = 23.34)	49.68 (SD = 21.24)	63.82 (SD = 23.69)	<.001
Injury Type, N (%)				<.001
Blunt	2,707 (87.4)	1,507 (82.7)	1,200 (94.1)	
Penetrating	389 (12.6)	314 (17.2)	75 (5.9)	
Burn	1 (0.0)	1 (0.1)	0 (0.0)	
Mechanism of Injury, N (%)				<.001
Fall	1,579 (51.0)	799 (43.9)	780 (61.2)	
MVC	754 (24.3)	437 (24.0)	317 (24.9)	
Motorcycle	165 (5.3)	144 (7.9)	21 (1.6)	
Pedestrian	159 (5.1)	94 (5.2)	65 (5.1)	
Assault	111 (3.6)	93 (5.1)	18 (1.4)	
Stab Wound	110 (3.6)	80 (4.4)	30 (2.4)	
Other	219 (7.1)	175 (9.6)	44 (3.5)	
Comorbidities, N (%) Hypertension Mental/Personality Disorders Diabetes Mellitus COPD Dementia Obesity Congestive Heart Failure Seizures Cerebral Vascular Accident Cancer Chronic Renal Failure Cirrhosis Parkinson’s Disease Myocardial Infarction Coronary Artery Disease Asthma Peripheral Arterial Disease Others	2,340 (75.6) 1,291 (41.7) 710 (22.9) 479 (15.5) 228 (7.4) 222 (7.2) 211 (6.8) 175 (5.7) 114 (3.7) 76 (2.5) 52 (1.7) 47 (1.5) 45 (1.5) 41 (1.3) 21 (0.7) 16 (0.5) 13 (0.4) 4 (0.1) 163 (5.3)	1,297 (71.2) 628 (34.5) 330 (18.1) 258 (14.2) 93 (5.1) 76 (4.2) 110 (6.0) 68 (3.7) 72 (4.0) 28 (1.5) 14 (0.8) 24 (1.3) 29 (1.6) 23 (1.3) 14 (0.8) 7 (0.4) 3 (0.2) 1 (0.1) 97 (5.3)	1,043 (81.8) 663 (52.0) 380 (29.8) 221 (17.3) 135 (10.6) 146 (11.5) 101 (7.9) 107 (8.4) 42 (3.3) 48 (3.8) 38 (3.0) 23 (1.8) 16 (1.3) 18 (1.4) 7 (0.5) 9 (0.7) 10 (0.8) 3 (0.2) 66 (5.2)	<.001 <.001 <.001 .016 <.001 <.001 .041 <.001 .339 <.001 <.001 .276 .411 .720 .464 .219 .009 .312 .857
Current Smoker, N (%)	762 (24.6)	532 (29.2)	230 (18.0)	<.001
Alcohol Use Disorder, N (%)	263 (8.5)	197 (10.8)	66 (5.2)	<.001
Substance Use Disorder, N (%)	129 (4.2)	84 (4.6)	45 (3.5)	.138
Anticoagulant Therapy, N (%)	347 (11.2)	184 (10.1)	163 (12.8)	.020
Steroid Use, N (%)	61 (2.0)	21 (1.2)	40 (3.1)	<.001

Abbreviations: MVC, motor vehicle collision; COPD, chronic obstructive pulmonary disease.

**Table 2.** Hospital Disposition Characteristics

Characteristics	Total	Male	Female	P Value
Post ED Disposition, N (%)				<.001
Floor	1,395 (45.0)	702 (38.5)	693 (54.4)	
Intensive Care Unit (ICU)	853 (27.5)	555 (30.5)	298 (23.4)	
Operating Room	581 (18.8)	404 (22.2)	177 (13.9)	
Not Applicable	157 (5.1)	85 (4.7)	72 (5.6)	
Home	44 (1.4)	31 (1.7)	13 (1.0)	
Morgue	38 (1.2)	33 (1.8)	5 (0.4)	
Observation Unit	22 (0.7)	9 (0.5)	13 (1.0)	
Labor and Delivery	4 (0.1)	0 (0.0)	4 (0.3)	
Acute Care Facility	1 (0.0)	1 (0.1)	0 (0.0)	
Correctional Facility	1 (0.0)	1 (0.1)	0 (0.0)	
Left Against Medical Advice	1 (0.0)	1 (0.1)	0 (0.0)	
Discharge Status, N (%)				.002
Alive	2,924 (94.4)	1,701 (93.4)	1,223 (95.9)	
Dead	173 (5.6)	121 (6.6)	52 (4.1)	
ICU Admission, N (%) Median Duration of ICU Stay, Days (IQR)	1210 (39.1) 3 (2-5)	792 (43.5) 3 (2-5)	418 (32.8) 3 (2-4)	<.001 .009
Ventilation, N (%) Median Duration of Ventilation, Days (IQR)	447 (14.4) 2 (1-7)	337 (18.5) 3 (1-8)	110 (8.6) 2 (1-6)	<.001 .042
Hospital Length-of-Stay (Days)	4 (2-8)	4 (2-8)	4 (2-8)	.227

Abbreviations: ED, emergency department; ICU, intensive care unit.

In order to determine the triage category (Immediate, Delayed, Minimal, Expectant, or Dead) using the studied triage systems (SALT and META), the investigators reviewed each case. As some of the triage systems required clinician decisions that were not directly captured in the trauma registry data, surrogate criteria were used to assign a triage category (Supplementary Table 1; available online only). Each patient was also manually categorized to a triage category by the study investigator using three proposed “gold standards” – Injury Severity Score (ISS), Revised Trauma Score (RTS), and the Lerner, et al consensus criteria.

The following “gold standards” of the RTS/Sort Triage, ISS, and Lerner criteria were used or calculated to determine the patient’s actual outcome. The RTS and Sort triage is determined by combining the score of Glasgow Coma Scale (GCS), respiratory rate, and systolic blood pressure. The total score ranges between 0 and 12. The RTS/Sort triage assigned the RTS score of 1-10 as Immediate (red), 11 as Delayed (yellow), 12 as Minimal (green), and 0 as Dead (black) category.^16,17^

The ISS is calculated by adding the squares of the top three Abbreviated Injury Scale (AIS) scores across six different body regions (the head and neck, face, chest, abdomen, limbs, and externals). The ISS ranges from 1-75. An increase in trauma patient mortality is correlated with a higher ISS score. A cutoff score of 15 is typically used to differentiate mild from severe trauma.^18^

The Consensus-Based Gold Standard for the Evaluation of Mass-Casualty Triage Systems (Lerner, et al) listed multiple criteria related to patient clinical findings and diagnoses to assign a patient’s “true” category.^1^ Not all criteria could be used due to the trauma registry’s data limitations. The Lerner criteria used in this study are listed in Supplementary Table 2 (available online only).

### Outcome Measures

The primary objective of this research was to evaluate the performance and statistical agreement of the META and SALT mass-casualty triage systems with various standards. Secondary objectives included examining whether patient outcomes, such as mortality, hospital length-of-stay, ICU admission, ED disposition, and blood product usage within 24 hours, differed based on MCI triage categories.

### Sample Size Determination

The sample size was estimated according to a formula for estimated prevalence and sensitivity.^19^ The expected sensitivity value referred from a previous study was defined SALT was 0.65^20^ and META was 0.799.^21^ The prevalence of trauma patients receiving the triage category Immediate was 0.04^4^; d was defined as 0.1; α = 0.05; and the necessary sample size was therefore 2,185. Because data were retrospectively collected from medical records, an additional 25% was calculated according to a sample size adjustment formula (*n*_new_ = 2185/[1 – 0.25]), which yielded 2,913.33. Therefore, the sample size needed for this study was set to be 3,000. The sample was chosen in accordance with simple random sampling among trauma patients from January 1, 2018 through December 31, 2020.

### Statistical Analysis

Continuous variables were presented as means and standard deviations (SD), or medians and interquartile ranges (IQR), while categorical variables were presented as frequencies and proportions. The two groups were compared using the independent t-test or Mann-Whitney U test for numeric variables and the chi-squared test or Fisher’s exact test for categorical variables.

The above data variables were compared between the MCI triage systems. With 95% confidence intervals (CI), each of the triage category assignments were analyzed to determine their sensitivity, specificity, and predictive values when compared to one of the selected “gold” standards. Cohen’s Kappa statistic was utilized to determine the agreement between each triage method and the standard systems (RTS/Sort triage and Lerner, et al consensus-based criteria). Correlation of each triage method to the ISS was determined using the Spearman correlation coefficient. If there were a significant correlation, further subgroup analysis would be conducted to determine what percentage of patients with an ISS of 15 or higher fell under the Immediate triage category. The association between each triage category and the secondary outcome indicators were analyzed using regression analysis.

Commercially-available statistics software (IBM SPSS Statistics for Windows, Version 26.0; IBM Corp.; Armonk, New York USA) was used for statistical analysis. All statistical tests were considered statistically significant if the P value was ≤.05.

### Ethical Statement

This study was conducted in accordance with the tenets of the Helsinki Declaration of 1975 and its revisions in 2000. This study was reviewed by the Institutional Review Board of the Medical College of Wisconsin and determined to be exempt (application PRO00043635). This study was also reviewed and approved by the Research Ethics Committee of the Ministry of Health of Asturias (Oviedo, Spain). The informed consent requirement was waived because of the retrospective nature and anonymity of all patient data.

## Results

### Patients’ General Characteristics

A total of 3,097 patients were randomly selected for this study. The average age was 55.5 years and 55.8% of patients were male. The most frequent type of injury was blunt injury (87.4%). The most common causes of injuries were falls (51%), motor vehicle collisions (24%), and motorcycle crashes (5.3%). There were statistically significant differences between sex in several of the characteristics (Table 1).

### Hospital Disposition Characteristics

There was a 5.6% overall mortality rate across all subjects. The rate of admission to the ICU was 39.1%. Median ICU length-of-stay was three days (IQR 2-5). A total of 581 cases (18.8%) were sent to emergency surgery from the ED. Median hospital length-of-stay was four days (IQR 2-8; Table 2). There were also differences between sex in several of the disposition categories.

### Triage Prioritization Categories

Most patients were categorized as Delayed in SALT triage (65.8%), META triage (55.4%), and Sort triage (7.5%). The category breakdown using consensus-based gold standard criteria: Immediate (14.8%), Delayed (83.8%), Minimal (0.7%), Expectant (0.5%), and Dead (0.3%); Table 3.

**Table 3.** Triage Categories

Triage Category/Color Code	SALT Triage		META Triage		Sort Triage		Consensus-Based Criteria	
	N	%	N	%	N	%	N	%
Immediate/Red	1,058	34.2	1,380	44.6	316	10.2	458	14.8
Delayed/Yellow	2,039	65.8	1,717	55.4	232	7.5	2,594	83.8
Minimal/Green					2,539	82.0	21	0.7
Expectant/Gray							16	0.5
Dead/Black					1	0.0	8	0.3
Not Applicable					9	0.3		

Abbreviations: SALT, Sort, Assess, Lifesaving Interventions, Treatment/Transport; META, Advanced Prehospital Triage Model (Modelo Extrahospitalario de Triaje Avanzado).

### Comparisons of META and SALT Triage versus Sort and Lerner Consensus Criteria

Both the RTS/Sort system and the Lerner consensus criteria were used to determine the test characteristics for the SALT and META triage systems. The results of the META and SALT triage methods using the two gold standard comparisons are shown in Table 4.

**Table 4.** Comparisons of META and SALT Triage versus Sort and the Lerner Consensus Criteria

Triage Category	Sensitivity		Specificity		PPV		NPV	
	%	95% CI	%	95% CI	%	95% CI	%	95% CI
META Triage Test Characteristics (Sort Triage as Gold Standard)								
Immediate	91.5	87.8-94.3	60.8	58.9-62.6	20.9	18.8-23.2	98.4	97.7-99.0
Delayed	28.9	23.1-35.2	42.4	40.6-44.2	3.9	3.0-4.9	88.0	86.2-89.7
SALT Triage Test Characteristics (Sort Triage as Gold Standard)								
Immediate	94.9	91.9-97.1	72.7	71.0-74.4	28.4	25.7-31.2	99.2	98.7-99.6
Delayed	1.3	0.3-3.7	28.9	27.3-30.6	0.1	0.0-0.4	78.4	75.7-80.8

**Triage Category**	**Sensitivity**		**Specificity**		**PPV**		**NPV**	
	%	95% CI	%	95% CI	%	95% CI	%	95% CI
META Triage Test Characteristics (Lerner Criteria as Gold Standard)								
Immediate	77.1	72.9-80.8	61.1	59.2-62.9	25.6	23.3-28.0	93.9	92.6-95.0
Delayed	61.4	59.5-63.3	75.1	71.1-78.9	92.7	91.4-93.9	27.4	25.1-29.8
SALT Triage Test Characteristics (Lerner Criteria as Gold Standard)								
Immediate	68.6	64.1-72.8	71.8	70.0-73.5	29.7	26.9-32.5	92.9	91.7-94.0
Delayed	72.2	70.5-74.0	67.2	62.9-71.3	91.9	90.6-93.1	31.9	29.1-34.9

Abbreviations: SALT, Sort, Assess, Lifesaving Interventions, Treatment/Transport; META, Advanced Prehospital Triage Model (Modelo Extrahospitalario de Triaje Avanzado); PPV, positive predictive value; NPV, negative predictive value.

### Prediction of Secondary Outcomes by META and SALT Triage

Prediction of secondary outcomes related to META and SALT triage results are presented in Table 5. Regarding discharge status (alive or dead), 10.5% of patients whom META triaged as Immediate died, compared to 1.6% of patients in the Delayed category. The median length-of-stay in the ICU for patients in the META Immediate category was three days (IQR 2-6) compared to three days (IQR 2-4) for patients in the Delayed category. The median length of mechanical ventilation was two days (IQR 1-8) for the META Immediate category and two days (IQR 1.5-5.5) for Delayed. The median length of hospital stay for META Immediate patients was five days (IQR 2-9), while the median length of hospital stay for META Delayed patients was four days (IQR 2-7). Red blood cells (RBCs) were given to patients with META’s Immediate category at a median of four units (IQR 2-9) compared to the Delayed group at a median of three units (IQR 2-4). Regarding ED disposition, for patients META triaged as Immediate, 32.5% were admitted to the hospital floor, 33.9% to the ICU, 25.7% to the operating room, 0.8% were sent home, and 2.8% were sent to the morgue. For patients who were META triaged as Delayed, 55.2% were admitted to the hospital floor, 22.4% admitted to the ICU, 13.2% to the operating room, and 1.9% were sent home. No patients were moved from the ED to the morgue under this category. For META, overall mortality, ICU length-of-stay, hospital length-of-stay, and ED disposition were all statistically different between the Immediate and Delayed categories.

**Table 5.** Prediction of Secondary Outcomes Related to META and SALT Triage

Outcomes	Immediate/Red N (%)		Delayed/Yellow N (%)		P Value
**Triage Category by META**					
Discharge Status					<.001
Alive	1,235	(89.5)	1,689	(98.4)	
Dead	145	(10.5)	28	(1.6)	
Post-ED Disposition					<.001
Floor	448	(32.5)	947	(55.2)	
Intensive Care Unit (ICU)	468	(33.9)	385	(22.4)	
Operating Room	355	(25.7)	226	(13.2)	
Not Applicable	54	(3.9)	103	(6.0)	
Home	11	(0.8)	33	(1.9)	
Morgue	38	(2.8)	0	(0.0)	
Observation Unit	4	(0.3)	18	(1.0)	
Labor and Delivery	2	(0.1)	2	(0.1)	
Acute Care Facility	0	(0.0)	1	(0.1)	
Correctional Facility	0	(0.0)	1	(0.1)	
Left AMA	0	(0.0)	1	(0.1)	
	Median	(IQR)	Median	(IQR)	
ICU Length-of-Stay (Days)	3	(2-6)	3	(2-4)	<.001
Ventilation (Days)	2	(1-8)	2	(1.5-5.5)	.311
Hospital Length-of-Stay (Days)	5	(2-9)	4	(2-7)	<.001
RBCs Received within First 24 Hours (Units)	4	(2-9)	3	(2-4)	.055
**Triage Category by SALT**					
**Outcomes**	**Immediate/Red N (%)**		**Delayed/Yellow N (%)**		**P Value**
Discharge Status (%)					
Alive	907	(85.7)	2,017	(98.9)	<.001
Dead	151	(14.3)	22	(1.1)	
Post-ED Disposition (%)					
Floor	291	(27.5)	1,104	(54.1)	<.001
ICU	438	(41.4)	415	(20.4)	
Operating Room	227	(21.5)	354	(17.4)	
Not Applicable	52	(4.9)	105	(5.1)	
Home	8	(0.8)	36	(1.8)	
Morgue	37	(3.5)	1	(0.0)	
Observation Unit	3	(0.3)	19	(0.9)	
Labor and Delivery	2	(0.2)	2	(0.1)	
Acute Care Facility	0	(0.0)	1	(0.0)	
Correctional Facility	0	(0.0)	1	(0.0)	
Left AMA	0	(0.0)	1	(0.0)	
	Median	(IQR)	Median	(IQR)	
ICU Length-of-Stay (Days)	3	(2-7)	3	(2-4)	<.001
Ventilation (Days)	3	(1-8)	2	(1-5)	.202
Hospital Length-of-Stay (Days)	5	(2-10)	4	(2-7)	<.001
RBCs Received within First 24 Hours (Units)	4	(2-10)	3	(2-5)	.007

Abbreviations: SALT, Sort, Assess, Lifesaving Interventions, Treatment/Transport; META, Advanced Prehospital Triage Model (Modelo Extrahospitalario de Triaje Avanzado); ED, emergency department; AMA, against medical advice; ICU, intensive care unit; RBC, red blood cell.

In total, 14.3% of patients identified by SALT in the Immediate category died as compared to 1.1% in the Delayed category. In the Immediate group, the median length-of-stay in the ICU was three days (IQR 2-7), whereas in the Delayed group, it was three days (IQR 2-4). Median mechanical ventilation time was three days (IQR 1-8) for the Immediate category and two days (IQR 1-5) for Delayed. In the Immediate group, the median hospital length-of-stay was five days (IQR 2-10) compared to the Delayed group’s four days (IQR 2-7). Patients who SALT categorized as Immediate received a median of four RBC units (IQR 2-10), whereas patients categorized as Delayed received a median of three units (IQR 2-5). In terms of how SALT-triaged patients in the ED were handled, 27.5% were moved to hospital floors, 41.4% were sent to ICUs, 21.5% were sent to surgery, 0.8% were sent home, and 3.5% were taken to the morgue. Patients who were SALT triaged as Delayed were admitted to hospital floors in 54.1% of cases, ICUs in 20.4%, operating rooms in 17.4%, and home in 1.8% of cases. Under the Delayed category, just one patient was transferred from the ED to the morgue. Mortality, ICU length-of-stay, hospital length-of-stay, units of RBCs received, and ED disposition were statistically different between the SALT Immediate and Delayed groups.

## Discussion

The study compared the META and SALT mass-casualty triage systems to selected gold standard systems using actual patient data from a trauma registry. Both META and SALT triage algorithms are more flexible and clinically based than traditional physiologic criteria-based triage methods. They assess patients’ ABCDEs as decision criteria. While META uses clinical judgment to determine if a patient has abnormal ABCDE status, SALT relies on physical exam to evaluate ABCDE status. Also, META triage establishes specific criteria for early surgical evaluation (Q), classifying patients with a specific injury mechanism and hemodynamic instability as falling under the Immediate category.^9,21^ This injury criterion is not present in the SALT algorithm. The differences in the decision-making process may account for some of the results noted in this study.

In MCI triage systems, it is important to reach a balance between sensitivity and specificity in order to efficiently use limited resources. Very high sensitivty can lead to over-triage, which would then allocate resources to patients that do not require them. Very high specificity can reduce the chance of over-triage, but could lead to the opposite, or under-triage, missing severe conditions that require rapid intervention. It is generally accepted in routine practice that under-triage be kept under five percent, while over-triage be kept under 35%.^22^ However, in mass-casualty situations, it could be argued that a lower over-triage rate and more permissive under-triage rate (ie, lower sensitivity, higher specificity) might be tolerated in order to save limited resources. There are no suggested target test characteristics for mass-casualty triage. Based on this results, META and SALT perform differently for different gold standards, different evaluation methods, and different priorities. The positive predictive value (PPV) for SALT Immediate category cases was better than META, while PPV for SALT Delayed category cases was poor compared to META. Also, META had better sensitivity for Immediate patients when the Lerner consensus standard was used. Therefore, both SALT and META may have tendencies for over- or under-triage. It is also unclear whether the slight differences between META and SALT found in this study (at least in the Immediate category) have any clinical or resource use implication for both triage systems.

### Using Sort Triage as a Standard for Comparison

The Sort triage includes patient assessment utilizing formal physiological parameters. This method does not check for any signs of anatomical injuries. This method is based on the RTS system developed by Champion, et al,^23^ which uses systolic blood pressure, respiratory rate, and GCS. Thus, Sort triage is simple but relatively rigid. When applied to the study population, Sort triage reveals that 10.2% of patients were categorized as Immediate, 7.5% as Delayed, and 82.0% as Minimal. When using Sort as the comparison standard, both SALT and META triage show high sensitivity to the Immediate category. Though SALT triage had higher sensitivity (META 91.5%, SALT 94.9%) and specificity (META 60.8%, SALT 72.7%,), however, both SALT and META triage show low sensitivity (META 28.9%, SALT 1.3%) and specificity (META 42.4%, SALT 28.9%) when identifying Delayed patients. Categorizing the Immediate patient by SALT and META triage has a fair Kappa statistic agreement (META 0.210, SALT 0.332), and SALT has a higher percent agreement (META 54.33%, SALT 62.61%). Nonetheless, there was poor agreement over the Delayed category. These significant differences between the number of Delayed and Minimal patients identified by SALT and META compared to Sort cause the low sensitivity, specificity, and poor kappa statistic agreement. With both the SALT and META systems, there are generally specific criteria and decision points for the Immediate categories, while the Delayed categories are left primarily to clinical judgement. This may explain why the systems appear more accurate with Immediate category patients compared to the Delayed category. These results also may have been affected by the fact that none of the patients from the trauma database were triaged as Minimal (for reasons described below). Future research with additional patient data may help to further clarify these results.

### Using ISS for Comparison

The ISS ranges from 1-75. A higher score is correlated with a rise in trauma patient mortality. A cutoff score of 15 on the ISS is typically used to differentiate between mild and severe injury.^18^ When comparing SALT and META to the ISS using the Spearman correlation coefficient, SALT triage reveals a higher correlation relationship (META 0.222, SALT 0.304), though neither had a high correlation with the ISS. When analyzing the subgroup of the Immediate category, both triage methods had statistically significant higher proportions with an ISS of 15 or above compared to an ISS below 15. Patients triaged as Immediate by META and SALT are, therefore, more likely to have an ISS score of 15 or higher. Again, the more specific Immediate category criteria in the two systems may explain these findings.

### Using Lerner Consensus-Based Criteria for Comparison

This consensus-based gold standard was introduced in 2014 by Lerner, et al specifically for the purpose of standardizing mass-casualty triage research and comparisons.^1^ Using the Lerner criteria on the study population led to 14.8% identifying as Immediate, 83.8% as Delayed, 0.7% as Minimal, 0.5% as Expectant, and 0.3% as Dead categories. To identify the Immediate category, META has higher sensitivity (META 77.1%, SALT 68.6%) but lower specificity than SALT (META 61.1%, SALT 71.8%). For the Delayed category, SALT triage shows higher sensitivity (META 61.4%, SALT 72.2%) but lower specificity compared to META (META 75.1%, SALT 67.2%). The Kappa statistics comparing META and SALT triage to the gold standard also show fair agreement. Although SALT triage has a higher percent agreement than META in both Immediate and Delayed triage categories (META 63.45% versus SALT 71.33% for Immediate, 63.61% versus 71.42% for Delayed).

### Prediction of Secondary Outcomes Related to Triage Categories

Comparing proportions from the Immediate and Delayed categories as triaged by META and SALT shows some significant results that share similar patterns between both systems. For both SALT and META, the triaged Immediate patients tend to have higher mortality, longer ICU length-of-stay, and longer hospital length-of-stay. In regards to post-ED disposition, Immediate category patients are more likely to be admitted to the ICU and/or sent to the operating room for an emergent procedure when compared to Delayed patients. These data suggest that both SALT and META are useful in differentiating more severe patients.

## Limitations

This study’s main limitation is its retrospective review using trauma registry data, which are not specifically designed for disasters and MCIs. Since most of the patients in the trauma registry were not from an actual disaster/mass casualty (though there was a mass-casualty event during the studied period), it is possible that mass-casualty triage performed in real life could vary from the assignments performed in a study. It is also unclear whether these results would apply to medical (non-trauma) events. It should be noted that, according to past studies, most MCI’s involve traumatic injuries,^2,24^ so these results may be applicable. While a trauma registry provides large numbers of cases for analysis, it lacks certain detailed information that are used by mass-casualty triage systems. As a result, surrogate criteria (described in the Methods section) had to be used to apply the triage systems to the study population. It is unclear how these assumptions may have affected the results of this study.

The study population has other characteristics that may affect generalizability of these results. The study patients did show differences in proportion of female versus male patients in several demographic categories, including age, mechanism of injury, and past medical history. Again, since there were no patients with a primarily non-traumatic presentation, it is unclear how these results would apply to mass casualties due to biological, chemical, or radiological incidents. In terms of the standards used to determine the “actual” triage category, determination was limited by what was coded in the database. There was no way to know if there were other procedures or diagnoses that were not entered, or if there were errors in the recorded data. The proportion of Immediate category patients in this database is higher, and the Minimal category is lower than what has been reported in other studies.^4^ This is because patients presenting to a trauma center via EMS would have already undergone field triage. That process would have reduced the number of Minimal or Dead patients in the study population. Numerous factors that can affect the utilization of mass-casualty triage systems. Examples include the individuals who will function as the triage officers, their degree of expertise, the number of triage officers, the average triage time per case, the acceptable over- and under-triage rate, and the pre-existing triage plan. Finally, it should be noted that, although different possible “gold standards” have been used, there currently is still not a single accepted standard. Thus, interpreting results from this study should be done with an understanding of these limitations.

## Conclusion

This study showed that META and SALT triage perform similarly well in identifying seriously injured trauma patients. Patients who are assigned to the Immediate categories by META and SALT triage are significantly more likely to experience secondary outcomes such as mortality, emergent surgery, ICU admission, longer ICU and total hospital stays, and receiving more RBC units compared to patients who are assigned to the Delayed categories. These data suggest that both systems may be utilized as triage methods in an MCI or disaster situation where trauma patients predominate. Comparison of triage performance according to sensitivity, specificity, and statistical agreement are a few of the elements considered in deciding whether to implement a triage system. More research in triage science is needed to help determine the generalizability and practicality of using various mass-casualty triage systems.

## Supporting information

Tiyawat et al. supplementary material 1 Tiyawat et al. supplementary material

Tiyawat et al. supplementary material 2 Tiyawat et al. supplementary material

## Supplementary Materials

To view supplementary material for this article, please visit https://doi.org/10.1017/S1049023X24000098
