# Supplementary material for: Comparative Analysis of META and SALT Disaster Triage in an Adult Trauma Population: A Retrospective Observational Study
Source: Prehosp Disaster Med. 2024 Feb 26;39(2):142–50. doi: 10.1017/S1049023X24000098 (PMC11035921; doi:10.1017/S1049023X24000098)
Supplement: Tiyawat et al. supplementary material 1 — Tiyawat et al. supplementary material [file S1049023X24000098sup001.docx]

***Supplementary table 1*** *– Surrogate Criteria Used for Triage System Decisions*

| **Triage criteria: SALT** | **Surrogate criteria** | **Result** |
| --- | --- | --- |
| Breathing? | Respiratory rate of 0 or assisted respiratory rate documented | No |
|  | Any documented respiratory rate | Yes |
| Obeys commands or makes purposeful movements? | Glasgow Coma Scale (GCS) less than 14 | No |
|  | GCS of 14 or 15 | Yes |
| Has peripheral pulse? | No pulse rate documented, or pulse rate of less than 30, or systolic blood pressure (SBP) less than 90 | No |
|  | Any pulse rate 31 or greater and SBP 90 or greater | Yes |
| Respiratory distress? | Pulse oximetry 90% or greater and EMS respiratory rate between 4-20 /minute | No |
|  | Pulse oximetry less than 90% or respiratory rate less than 4 or greater than 20 | Yes |
| Major hemorrhage is controlled? | Was not used for categorization, as it was not possible to determine this given the limitations of the trauma registry data |  |
| Minor injuries only? | The investigators assumed that any patient presenting to the hospital as a trauma activation (and thus included in the hospital’s trauma registry) would be triaged as at least Delayed because they required hospital evaluation to rule-out any injuries. | No |
|  |  |  |
| **Triage criteria: META** | **Surrogate criteria** | **Result** |
| Actual or potential risk in airway? (A) | Any documented artificial airway/potential airway obstruction, or airway injuries or GCS of 8 or lower | Yes |
|  | No documented airway/airway obstruction and GCS 9 or higher | No |
| Actual or potential risk in breathing? (B) | Respiratory rate of 0 or apnea documented or assisted respiratory rate documented or pulse oximetry of 94% or lower | Yes |
|  | Spontaneous respiratory rate documented and pulse oximetry greater than 94% | No |
| Actual or potential risk in circulation? (C) | SBP less than 110 mmHg | Yes |
|  | Bleeding status was not used for categorization, as it was not possible to determine this given the limitation of the trauma registry data |  |
| Actual or potential risk in disability? (D) | Any documented GCS of 14 or lower | Yes |
|  | GCS of 15 | No |
| Actual or potential risk in exposure? (E) and require medical evaluation? | The investigators assumed that any patient presenting to the hospital as a trauma activation (and thus included in the hospital’s trauma registry) would be triaged as at least Delayed because they required hospital evaluation to rule-out any injuries | Yes |
