# Supplementary material for: Comparative Analysis of META and SALT Disaster Triage in an Adult Trauma Population: A Retrospective Observational Study
Source: Prehosp Disaster Med. 2024 Feb 26;39(2):142–50. doi: 10.1017/S1049023X24000098 (PMC11035921; doi:10.1017/S1049023X24000098)
Supplement: Tiyawat et al. supplementary material 2 — Tiyawat et al. supplementary material [file S1049023X24000098sup002.docx]

***Supplementary table 2*** *– Lerner Consensus Criteria*

| **Triage category** | **Criteria** |
| --- | --- |
| Expectant | In patients age 0 to 49 years old: third degree (full thickness) burns to >90% of the body |
|  | In patients over 50 years old: third degree (full thickness) burns to >80% of the body |
|  | Penetrating trauma to the head that crosses the midline with agonal respirations and/or no motor response, decorticate posturing, or decerebrate posturing |
|  | Blunt trauma to the head with agonal respirations and/or no motor response, decorticate posturing, or decerebrate posturing |
| Immediate | Neurologic, vascular, or hemorrhage-controlling surgery to the head, neck, or torso performed |
|  | Limb-conserving surgery performed |
|  | Escharotomy performed |
|  | Chest tube placed |
|  | An advanced airway intervention performed |
| Minimal | Discharge from the ED with no x-rays or extremity x-ray that was negative or showed an uncomplicated fracture; received only simple wound repair |
| Delayed | Any patient not meeting the above criteria for expectant, immediate, or minimal |
